# Real-world clinical outcomes of patients with CLN2 disease treated with cerliponase alfa

**DOI:** 10.3389/fneur.2025.1516026

**Published:** 2025-03-14

**Authors:** Angela Schulz, Christoph Schwering, Eva Wibbeler, Lena Marie Westermann, Luca Hagenah, Susanne Lezius, Ashok Jha, Abigail Hunt, Peter Slasor, Pascal Reisewitz, Miriam Nickel

**Affiliations:** ^1^Department of Pediatrics, University Medical Center Hamburg-Eppendorf, Hamburg, Germany; ^2^German Center for Child and Adolescent Health (DZKJ) Hamburg Partner Site, Hamburg, Germany; ^3^Institute of Medical Biometry and Epidemiology, University Medical Center Hamburg-Eppendorf, Hamburg, Germany; ^4^BioMarin Pharmaceutical Inc., Novato, CA, United States

**Keywords:** cerliponase alfa, CLN2, effectiveness, safety, real-world data, DEM-CHILD

## Abstract

**Introduction:**

This study assessed the real-world effectiveness and safety of the enzyme replacement therapy (ERT), cerliponase alfa, to treat neuronal ceroid lipofuscinosis type 2 (CLN2) disease.

**Methods:**

Data from the DEM-CHILD database were analyzed, comparing patients who initiated ERT outside clinical trials with natural history (NH) controls. Treated patients were matched 1:1 with NH controls on baseline age and combined motor-language (ML) score on the CLN2 clinical rating scale. Rate of ML score decline, time to unreversed 2-point decline or score of 0, and time to unreversed score of 0 were assessed. Safety was assessed in treated patients.

**Results:**

Twenty-four ERT-treated patients were eligible (mean [SD] follow-up: 106.7 [64.1] weeks); 21 matched to a NH control, with baseline mean (SD) age of 4.7 (1.9) years and mean (SD) ML score of 3.9 (1.6) points. ERT-treated patients had reduced likelihood of an unreversed ML 2-point decline or score of 0 (HR 0.08; 95% CI 0.02, 0.28; *p* < 0.0001), and unreversed ML score of 0 (HR 0.07; 95% CI 0.01, 0.40; *p* = 0.003) versus NH controls. Mean (SD) rate of ML score decline was 0.46 (0.43) versus 1.88 (1.45) points/48 weeks for ERT-treated and NH groups, respectively (mean difference: 1.42; 95% CI 0.74, 2.10; *p* = 0.0003). Sixteen patients (67%) had treatment-related adverse events; the most common were pyrexia (50%), vomiting (33%), and nausea (21%). No ERT-treated patients died.

**Discussion:**

Cerliponase alfa for real-world CLN2 disease treatment slowed decline in motor and language function and demonstrated an acceptable safety profile.

## 1 Introduction

Neuronal ceroid lipofuscinosis type 2 (CLN2) disease is a very rare, severe, neurodegenerative lysosomal storage disorder caused by autosomal recessive inheritance of genetic variants in the *TPP1* gene, which encodes for the lysosomal serine protease tripeptidyl peptidase 1 (1–4). Deficient tripeptidyl peptidase 1 activity leads to intralysosomal accumulation of autofluorescent storage material and is associated with neuronal and retinal cell loss (1–4). CLN2 disease typically presents with delayed language development and seizure onset in children aged 2–4 years. These symptoms are followed by rapidly progressing psychomotor decline, dementia, vision loss, and premature death, typically between 6 years of age and mid-adolescence (1, 2, 4, 5).

Early diagnosis is important for effective management of CLN2 disease. While clinical diagnosis has historically been delayed, often due to non-specific initial symptoms which could be commonly misinterpreted (4–6), early use of enzymatic and genetic testing may shorten the diagnostic journey and allow earlier intervention (7, 8). Until recently, the management of CLN2 disease was limited to symptomatic and palliative care (8, 9). In 2017, the US Food and Drug Administration and the European Medicines Agency approved cerliponase alfa, a recombinant human intracerebroventricular tripeptidyl peptidase 1 enzyme replacement therapy (ERT), as the first disease-modifying therapy for CLN2 disease (10, 11). Under EU approval, cerliponase alfa is indicated for the treatment of CLN2 disease in all ages (11). The initial US approval indicated treatment in symptomatic children 3 years of age and older; however, a recent update expanded the US indication to include children of all ages with CLN2 disease, regardless of symptom status (10).

The efficacy and safety of cerliponase alfa were evaluated in children and adolescents with CLN2 disease (aged 3–15 years) in the pivotal 48-week, phase 1/2 190-201 study (NCT01907087) and an open-label extension study (190-202; NCT02485899) for up to 240 weeks of follow-up (12, 13), and in the 190-203 study (NCT02678689), which assessed safety and efficacy in an expanded cohort that included children under 3 years of age. Patients’ baseline function and disease progression during these clinical studies were measured using the disease-specific CLN2 clinical rating scale (14), assessing combined scores in the motor and language domains. Outcomes for ERT-treated patients were compared with untreated natural history (NH) controls with CLN2 disease enrolled in the DEM-CHILD international neuronal ceroid lipofuscinosis (NCL) database, a clinical registry that enrolls children with different forms of NCL, including CLN2 disease (5, 6). ERT-treated patients and untreated NH controls were matched 1:1 on baseline criteria. In these trials, cerliponase alfa (300 mg every 2 weeks) was shown to effectively slow the otherwise rapid decline in motor and language function in children with CLN2 disease compared with untreated NH controls and was generally well tolerated, demonstrating an acceptable safety profile (12, 13).

Since the availability of cerliponase alfa, the DEM-CHILD database has collected data from patients with CLN2 disease who have received cerliponase alfa in clinical practice. This analysis used data in both treated and untreated patients from the DEM-CHILD database to assess the real-world safety and effectiveness of cerliponase alfa in children with CLN2 disease who initiated treatment outside of the clinical trial setting compared with NH controls.

## 2 Methods

### 2.1 Study design and data source

The DEM-CHILD database is a multicenter, multinational clinical registry that collects clinical, laboratory, imaging, and developmental information from patients with NCL disorders. This real-world, observational data analysis was based on data collected in the DEM-CHILD database, specifically from children with CLN2 disease (5). Data were prospectively collected from patients treated with cerliponase alfa between August 26, 2016 and December 31, 2020, while some data from untreated NH controls were collected retrospectively. This study was approved by the medical ethics committee of the Ärztekammer Hamburg, Germany (PV7215). Patients or parents provided consent for evaluation of data according to the tenets of the Declaration of Helsinki.

### 2.2 Patients and treatment

All patients included in this analysis had a confirmed diagnosis of CLN2 disease and ≥ 6 months of available follow-up data in the DEM-CHILD database (two motor-language [ML] score readings ≥ 6 months apart). Cerliponase alfa-treated (ERT-treated) patients were those who initiated cerliponase alfa 300 mg (or age-appropriate dose), administered once every other week as an intracerebroventricular (ICV) infusion, outside of the clinical trial setting. Untreated NH controls included in this analysis had never received cerliponase alfa.

### 2.3 Outcomes and assessments

The Hamburg scale is a four-item instrument developed to quantify the decline that occurs during the clinical course of CLN2 disease across four domains: motor, language, vision, and seizures (15). Each domain on this scale measures loss of function, where a score of 3 indicates normal condition and a score of 0 represents complete loss of function, with the total score ranging from 0 to 12 points. The ML domains of the Hamburg scale were subsequently adapted in the CLN2 clinical rating scale to be used as an assessment tool for clinical trials of cerliponase alfa (14) (see Supplementary Table S1). In this analysis, baseline function and CLN2 disease progression were assessed using the ML score, which were assessed at clinic visits every 6 months by the treating physician.

Adverse events (AEs) assessed by the treating physician to be drug or device related were assessed for patients in the ERT-treated cohort.

### 2.4 Matching and follow-up

For the primary analysis, ERT-treated patients were matched 1:1 with untreated NH controls based on two criteria: baseline age (± 12 months) and baseline ML score (exact match). An additional sensitivity analysis was performed in which ERT-treated patients were matched with controls based on three criteria: baseline age (± 12 months), baseline ML score (exact match), and genotype (0, 1, or 2 common alleles [c509-1G > C and c.622C > T]) (16). For ERT-treated patients, baseline ML score measurement was defined as the last observation before the first administration of cerliponase alfa; for NH controls, the baseline ML score was the assessment at the age of matching.

For ERT-treated patients, outcomes were analyzed from their individual baseline assessment until the last available follow-up assessment before the cut-off date for the analysis (December 31, 2020). Data from NH control patients were analyzed from their individual baseline assessment over a follow-up period equivalent in duration to that of the matched ERT-treated patient (i.e., follow-up assessments over the longest time period less than or equal to the duration of the matched ERT-treated patient were included).

### 2.5 Statistical analyses

Baseline demographic and clinical characteristics of the eligible and matched cohorts are summarized as means, standard deviations (SDs), medians, ranges, counts, and proportions, as dictated by data type. Baseline characteristics were compared between groups using *t*-tests for continuous variables and chi-square tests for categorical variables.

Kaplan–Meier methods and Cox proportional hazards models were used to compare the time to unreversed 2-point decline or a score of 0 and the time to unreversed score of 0 on the combined ML domains for ERT-treated patients and NH controls. In these models, ERT-treated patients who discontinued cerliponase alfa were not censored from the analyses. The Cox model included baseline ML score and baseline age as continuous covariates, and genotype (number of alleles) and sex as categorical covariates.

The rate of decline in ML score was calculated as the change from baseline to last assessment with a score of more than 0 divided by the length of follow-up (expressed as decline per 48-week period). The rates of decline were compared for ERT-treated patients and NH controls using a two-sided paired t-test, with unequal variance.

Combined ML scores by age were plotted for individually matched ERT-treated and NH patient pairs.

## 3 Results

### 3.1 Patients

A total of 24 patients who initiated cerliponase alfa treatment between August 2016 and May 2020 were eligible for inclusion (see Supplementary Table S2). The mean (SD) follow-up time for all ERT-treated patients was 106.7 (64.1) weeks. One ERT-treated patient discontinued treatment 12 weeks before the end of follow-up (the reason for discontinuation was not recorded).

Twenty-one ERT-treated patients could be matched with an NH control for the primary analysis; three ERT-treated patients were unable to be matched with an equivalent NH control patient on baseline ML score and baseline age. The mean (SD) follow-up time was 104.3 (63.9) weeks for the matched ERT-treated patients and 76.1 (43.1) for the matched NH control patients. Baseline demographic and clinical characteristics were generally similar for the matched ERT-treated and NH control patients, apart from sex (summarized in Table 1): baseline mean (SD) age was 4.7 (1.9) years and mean (SD) ML score was 3.9 (1.6) for both ERT-treated patients and NH controls.

**Table 1:** Baseline demographic and clinical characteristics of NH and ERT-treated patients (two-criteria match).

		NH (*N* = 21)	ERT-treated (*N* = 21)	*p*-value
Sex, *n* (%)	Female	5 (24)	11 (52)	0.06
	Male	16 (76)	10 (48)	
Age at baseline, years	Mean (SD)	4.7 (1.9)	4.7 (1.9)	0.96
	Median (min, max)	4.5 (0.7, 9.9)	4.5 (0.7, 9.9)	
Baseline ML score	Mean (SD)	3.9 (1.6)	3.9 (1.6)	1.00
	Median (min, max)	4.0 (1.0, 6.0)	4.0 (1.0, 6.0)	
Baseline ML score category, *n* (%)	1	1 (5)	1 (5)	
	2	4 (19)	4 (19)	
	3	3 (14)	3 (14)	
	4	6 (29)	6 (29)	
	5	2 (10)	2 (10)	
	6	5 (24)	5 (24)	
Phenotype^a^, *n* (%)	Typical	0	17 (81)	NA
	Atypical	0	3 (14)	
	Presymptomatic	0	1 (5)	
	NA	21 (100)	0	
Genotype, *n* (%)	2 common alleles^b^	13 (62)	14 (67)	0.93
	1 common allele	5 (24)	4 (19)	
	0 common alleles	3 (14)	3 (14)	
Age at disease onset, years	Mean (SD)	3.0 (0.8)	*n* = 20 3.4 (0.8)	0.10
	Median (min, max)	3.0 (1.3, 4.4)	3.3 (2.1, 6.0)	
Age at diagnosis, years	Mean (SD)	*n* = 17 5.2 (1.6)	4.2 (2.0)	0.07
	Median (min, max)	4.8 (2.9, 9.8)	3.9 (0.2, 8.8)	
First symptom^c^, *n* (%)	Seizures	13 (62)	17 (81)	0.17
	Language difficulties	9 (43)	13 (62)	0.22
	Motor difficulties	8 (38)	4 (19)	0.17
	Behavioral abnormalities	4 (19)	1 (5)	0.15
	Dementia	1 (5)	1 (5)	1.00
	Learning difficulties	1 (5)	1 (5)	1.00
	Vision loss	1 (5)	0	0.31
	Other/unknown	1 (5)	1 (5)	NA

a Phenotype was determined by physician adjudication.

b The common alleles were c.622C > T and c.509-1G > C.

c Patients may have had more than one presenting symptom.

Patients matched on baseline age (± 12 months) and baseline ML score. ERT, enzyme replacement therapy; ML, motor-language; NA, not assessed; NH, natural history; SD, standard deviation.

Baseline demographic and clinical characteristics of the matched ERT-treated population generally indicated it was heterogeneous for disease severity, including some patients with atypical phenotypes (*n* = 3 [14%]). The population included patients with normal baseline ML score (ML score = 6; *n* = 5 [24%]) and those with highly impaired ML function, indicating more advanced disease (ML score ≤ 2; *n* = 5 [24%]) (Table 1).

### 3.2 Effectiveness outcomes

**Figure 1:**
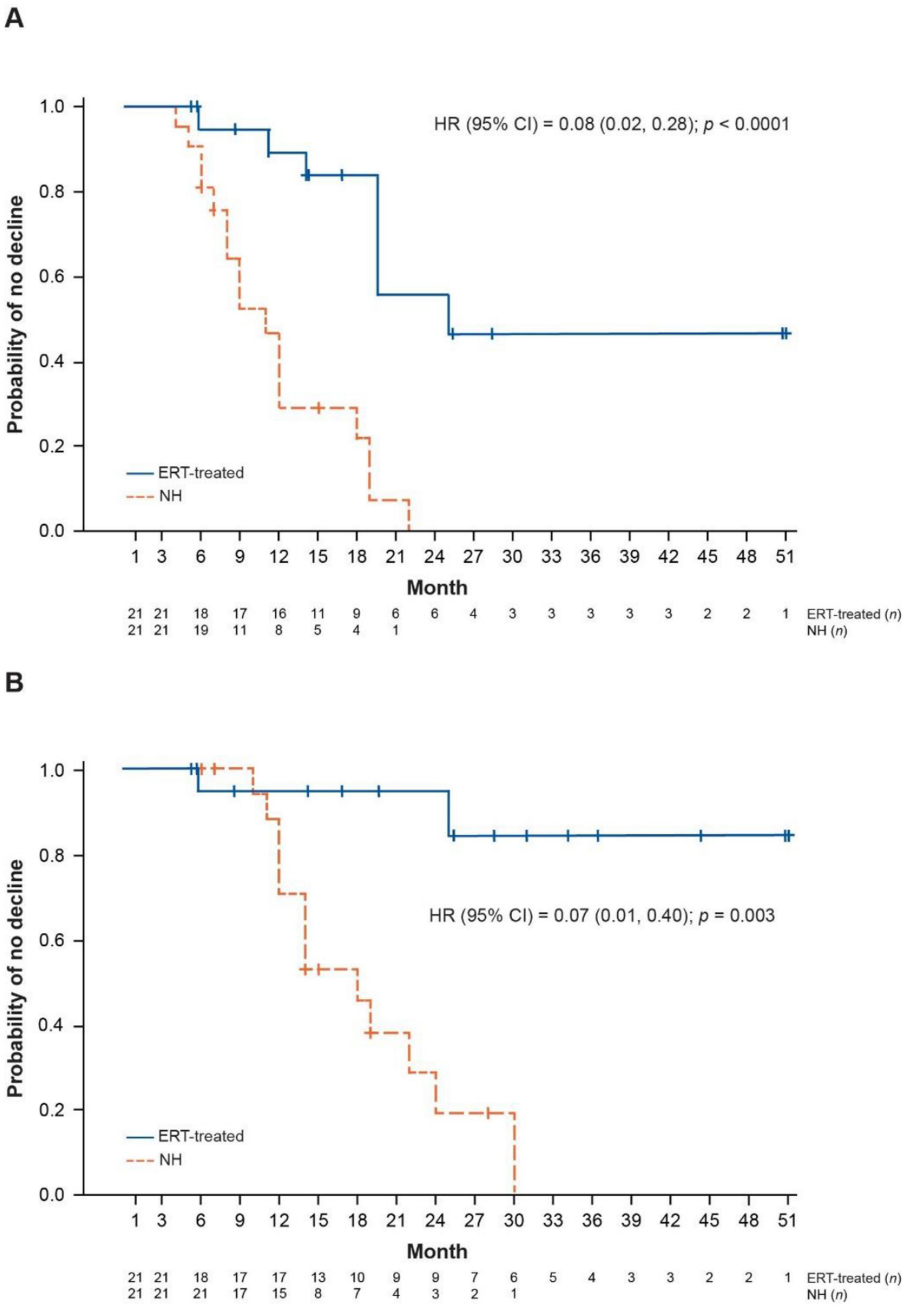
Kaplan–Meier plots of ML score outcomes for NH and ERT-treated patients (two-criteria match). **(A)** Time to unreversed 2-point decline or score of 0 in ML domains. **(B)** Time to unreversed score of 0 in ML domains. Patients matched on baseline age (± 12 months) and baseline ML score (exact match). CI, confidence interval; ERT, enzyme replacement therapy; HR, hazard ratio; ML, motor-language; NH, natural history.

Compared with NH controls, ERT-treated patients were found to be significantly less likely to have an unreversed ML 2-point decline or score of 0 (hazard ratio [HR] 0.08; 95% confidence interval 0.02, 0.28; *p* < 0.0001) (Figure 1A), or an unreversed ML score of 0 (HR 0.07; 95% confidence interval 0.01, 0.40; *p* = 0.003) (Figure 1B).

The rate of decline in ML score was significantly lower in ERT-treated patients: the mean (SD) rate of decline in ML score was 0.46 (0.43) points per 48 weeks among ERT-treated patients compared with 1.88 (1.45) points per 48 weeks among NH controls, with a mean (standard error) difference of 1.42 (0.33) (95% confidence interval 0.74, 2.10; *p* = 0.0003) (Table 2). When analyzed separately, the rate of decline in both motor and language domain scores was significantly lower in the ERT-treated group compared with NH controls (Table 2).

**Table 2:** Rate of decline in combined ML score and individual motor and language domain scores for NH and ERT-treated patients (two-criteria match).

	NH (*N* = 21)	ERT-treated (*N* = 21)
Rate of decline in ML score, points per 48-week period		
*n*	21	21
Mean (SD)	1.88 (1.45)	0.46 (0.43)
Median (min, max)	1.66 (0.00, 5.60)	0.44 (0.00, 1.33)
95% CI	1.22, 2.54	0.26, 0.65
Mean (SE) difference	1.42 (0.33)	
95% CI	0.74, 2.10	
*p*-value	0.0003	
Rate of decline in motor score, points per 48-week period		
*n*	20	20
Mean (SD)	0.99 (0.75)	0.23 (0.28)
Median (min, max)	1.02 (0.00, 2.40)	0.11 (0.00, 0.79)
95% CI	0.64, 1.34	0.10, 0.36
Mean (SE) difference	0.75 (0.18)	
95% CI	0.38, 1.12	
*p*-value	0.0003	
Rate of decline in language score, points per 48-week period		
*n*	19	18
Mean (SD)	1.04 (0.96)	0.16 (0.28)
Median (min, max)	0.80 (0.00, 3.73)	0.00 (0.00, 0.72)
95% CI	0.58, 1.50	0.02, 0.30
Mean (SE) difference	0.88 (0.23)	
95% CI	0.40, 1.36	
*p*-value	0.0010	

Patients matched on baseline age (± 12 months) and baseline ML score (exact match). CI, confidence interval; ERT, enzyme replacement therapy; ML, motor-language; NH, natural history; SD, standard deviation; SE, standard error.

Patient-level observations of ML score over follow-up generally demonstrated stabilization or slower decline in ML score for patients receiving cerliponase alfa compared with their matched NH control. Results were consistent across age at treatment initiation (< 3 years to ≥6 years) and baseline ML score (see Supplementary Figure S1). At the end of follow-up, the ERT-treated patient had a greater ML score than the NH control in 15/21 matched pairs (71%) and an equivalent ML score to the NH control in 6/21 matched pairs (29%) (see Supplementary Figure S1).

In the five matched pairs with baseline ML scores of ≤ 2, cerliponase alfa showed a comparative treatment benefit in three patients: one maintained their ML score and two showed slower disease progression versus their matched NH control over follow-up. The remaining two matched patients had no comparative benefit with cerliponase alfa on ML score decline.

A sensitivity analysis using three-criteria patient matching (on the basis of baseline age, ML score, and genotype) provided similar results for the rate of decline in ML score per 48-week period, the time to unreversed 2-point decline or ML score of 0, and to unreversed ML score of 0, as compared with matched NH controls (see Supplementary Tables S3 and S4, Supplementary Figures S2 and S3).

### 3.3 Treatment-related AEs

Seventy-five treatment-related AEs were observed in 16/24 (67%) ERT-treated patients (Table 3). Most treatment-related AEs were moderate in severity (Grade 2, *n* = 14 [58%]); four patients (17%) had an AE of Grade 3 or higher. The most common treatment-related AEs were pyrexia (50%), vomiting (33%), and nausea (21%). Seven patients had 19 treatment-related AEs resulting in hospitalization; three device-related infections occurred in three patients (13%), and two device leakages occurred in two patients (8%).

**Table 3:** Summary of treatment-related AEs in all ERT-treated patients.

	All treatment-related AEs (*N* = 24)			Treatment-related AEs resulting in hospitalization (*N* = 24)		
	Incidence, *n* (%)	Events	Rate, events/100 pt-years	Incidence, *n* (%)	Events	Rate, events/100 pt-years
Any treatment-related AE	16 (66.7)	75	13.40	7 (29.2)	19	3.39
Grade 2	14 (58.3)	63	11.25	3 (12.5)	7	1.25
Grade 3	1 (4.2)	1	0.18	1 (4.2)	1	0.18
Grade 4	3 (12.5)	11	1.96	3 (12.5)	11	1.96
Treatment-related AEs						
Pyrexia	12 (50.0)	43	7.68	4 (16.7)	5	0.89
Vomiting	8 (33.3)	10	1.79	5 (20.8)	6	1.07
Nausea	5 (20.8)	5	0.89	2 (8.3)	2	0.36
Arrythmia	3 (12.5)	3	0.54	0	0	0
Device-related infection	3 (12.5)	3	0.54	3 (12.5)	3	0.54
Seizure	3 (12.5)	6	1.07	0	0	0
Device leakage	2 (8.3)	2	0.36	2 (8.3)	2	0.36
Flushing	1 (4.2)	2	0.36	0	0	0
Headache	1 (4.2)	1	0.18	1 (4.2)	1	0.18

AE, adverse event; ERT, enzyme replacement therapy; pt, patient.

No ERT-treated patients died during the follow-up period, whereas six NH patients (29%) in the matched cohort died over the equivalent period (median time to death: 313 weeks).

## 4 Discussion

Understanding the benefit of ERT for patients with CLN2 disease in a real-world setting is an important clinical question. The data presented here are based on the largest cohort of patients with CLN2 disease receiving cerliponase alfa treatment outside of a clinical trial setting. Using analyses analogous to those used in the cerliponase alfa 190-201/202 clinical trials (12, 13), this study demonstrated that treatment with cerliponase alfa significantly slowed the deterioration in motor and language function compared with untreated NH controls in the real-world setting. Moreover, treatment-related AEs were generally consistent with those observed in the 190-201/202 clinical trials (12, 13), with no new safety signals identified.

The results observed here are consistent with the efficacy findings from the 190-201/202 clinical trials of cerliponase alfa (12, 13). In the current analysis, mean difference in the rate of ML score decline per 48 weeks between ERT-treated patients and NH controls was similar to that reported in the clinical trials (mean difference: 1.42 vs. 1.75 points) (13). Furthermore, observations that ERT-treated patients compared with NH controls were at significantly lower risk of an unreversed 2-point decline or ML score of 0 (HR 0.08) and an unreversed ML score of 0 (HR 0.07) are consistent with findings from the clinical trials (HR 0.14 and 0.02, respectively) (12, 13).

The DEM-CHILD cohort represents a more heterogeneous population than the clinical trial population, including presymptomatic patients, those with atypical phenotypes, and patients at advanced stages of ML score impairment. Patients enrolled in the clinical trials were required to have an ML score between 3 and 6 and a score of at least 1 on each domain at screening (although at time of first dose, scores ranged from 1 to 6). While the mean baseline ML score in this DEM-CHILD cohort was similar to that recorded for the clinical trial population (mean ML score: 3.9 vs. 3.7), the DEM-CHILD cohort had a larger proportion of patients with lower ML scores at baseline (24% [*n* = 5] vs. 0% with ML score ≤ 2) (12). The present study provided an opportunity to explore the effectiveness of cerliponase alfa in patients with more advanced disease at treatment initiation. There was some indication of treatment benefit on ML score deterioration observed in three patients with baseline ML score ≤ 2; however, benefit was not observed in the remaining two ERT-treated patients with baseline ML score ≤ 2. Further evidence is required to determine the effectiveness of cerliponase alfa in patients with advanced disease at treatment initiation.

Of the five patients who initiated treatment with cerliponase alfa before deterioration of motor and language function (ML score = 6), three retained an ML score of 6 over their respective durations of follow-up (7, 8, and 10 months). However, it should be noted that these patients had not reached the age at which the most significant decline would be expected based on the natural history of the classical CLN2 disease phenotype by the end of follow-up (1, 4, 5). The remaining two patients demonstrated an initial decline shortly after treatment initiation, followed by a relative stabilization in ML score versus their matched NH control, over their respective durations of follow-up (13 and 35 months). Longer follow-up will be required to confirm these findings, which may suggest a clinically relevant benefit for very early intervention in children diagnosed with CLN2 disease.

No new safety signals were identified, and the profile of treatment-related AEs observed was generally consistent with those recorded during clinical trials (12, 13). The incidence of ICV device-related infections observed was generally comparable to or lower than those reported during clinical trials, in which nine patients (38%) had device-related infections over > 5 years of treatment (13), and two patients (8%) had eight device leakages (12). Given the high frequency of ICV infusions (minimum of 25 per year), strict adherence to best practice guidelines is critical to maintain low device-related infection and AE rates, ultimately improving patient safety and reducing the risk of treatment interruption or discontinuation (17).

There were no deaths in this cohort of ERT-treated patients during the duration of follow-up, whereas six of the matched untreated NH controls died over the equivalent period. In the natural history of CLN2 disease, the median age at death has been reported to be 10.0 years (5); therefore, the early initiation of cerliponase alfa is important to attenuate disease progression and prolong life (8). Managing the symptoms of CLN2 disease (e.g., via the use of feeding tubes, and/or mechanical ventilators) may reduce disease burden and increase life expectancy (8, 9). However, such measures generally do not slow the characteristic progression of the disease. By contrast, cerliponase alfa has been shown to modify disease progression, extending the period that patients remain healthy. This can ultimately delay requirements for end-of-life care, which is associated with substantial physical and emotional strain for patients, families, and caregivers (8, 18).

This analysis does have some limitations, most notably the lack of a contemporaneous control group: since the approval of cerliponase alfa there have been very few untreated patients, and comparisons in this study were made using the best and most comprehensive historical controls available. Additionally, despite the use of matching on baseline age, baseline ML score (two-criteria match), and genotype (three-criteria match), the potential for residual confounding factors remains. Furthermore, only AEs considered to be treatment/ICV device related were recorded, and so there was potential for reporting bias. The sample size for these analyses was small (*n* = 24), although the cohort is quite substantial for an ultra-rare disease; previous single-center analyses of cerliponase alfa have reported similar-sized cohorts (17, 19). There were also limited follow-up data for some patients, particularly those aged < 3 years at baseline. Additionally, although the ERT-treated cohort included patients with both typical and atypical phenotypes, patient numbers in the atypical phenotype subgroup were small (*n* = 3 [14%]), and this analysis did not specifically evaluate the effectiveness of ERT in those patients. Future analyses of the DEM-CHILD cohort will be important to evaluate the impact of ERT on slowing disease progression in patients with atypical phenotypes.

In conclusion, cerliponase alfa was found to significantly slow the deterioration in motor and language function in children with CLN2 disease treated in real-world clinical practice. These results align with efficacy and safety findings from clinical trials. Future analyses of the DEM-CHILD database with longer follow-up will be vital to evaluate the benefits of very early treatment initiation in presymptomatic patients, as well as the impact of long-term treatment with cerliponase alfa on disease trajectory, including the assessment of seizures, movement disorders, quality of life and other patient- or caregiver-reported outcome measures, and lifespan. Additionally, the heterogeneity of the DEM-CHILD cohort presents an opportunity to explore research questions beyond those evaluated in the clinical trial population, including the impact of ERT in patients with atypical phenotypes and those with more advanced stages of baseline ML score impairment.

## Data Availability

De-identified individual participant data that underlie the results reported in this article (including text, tables, figures, and appendices) will be made available together with the research protocol and data dictionaries, for non-commercial, academic purposes. Additional supporting documents may be available upon request. Investigators will be able to request access to these data and supporting documents via a data sharing portal. Requests must include a research proposal clarifying how the data will be used, including proposed analysis methodology. Access will be given, contingent upon execution of a data access agreement with University Medical Center Hamburg-Eppendorf.
