# Supplementary material for: Real-world clinical outcomes of patients with CLN2 disease treated with cerliponase alfa
Source: Front Neurol. 2025 Mar 14;16:1516026. doi: 10.3389/fneur.2025.1516026 (PMC11951263; doi:10.3389/fneur.2025.1516026)
Supplement: Supplementary file 1 [file Supplementary_file_1.docx]

**Supplementary Material**

**TABLE S1 The Hamburg scale and CLN2 CRS ML Domain items.**

| **Domain** | **Score** | **Hamburg scale** | **CLN2 CRS** |
| --- | --- | --- | --- |
| Motor | 3 | Walks normally | Grossly normal gait; no prominent ataxia, no pathologic falls |
|  | 2 | Frequent falls, clumsiness obvious | Independent gait as defined by ability to walk without support for 10 steps; will have obvious instability and may have intermittent falls |
|  | 1 | No unaided walking or crawling only | Requires external assistance to walk or can only crawl |
|  | 0 | Immobile, mostly bedridden | Can no longer walk or crawl |
| Language | 3 | Normal (individual maximum) | Apparently normal language. Intelligible and grossly age appropriate. No decline noted yet |
|  | 2 | Has become recognizably abnormal | Language has become recognizably abnormal: some intelligible words; may form short sentences to convey concepts, requests, or needs. This score signifies a decline from a previous level of ability (from the individual maximum reached by the child) |
|  | 1 | Hardly understandable | Hardly understandable, few intelligible words |
|  | 0 | Unintelligible or no language | No intelligible words or vocalizations |

CLN2, neuronal ceroid lipofuscinosis type 2; CRS, clinical rating scale; ML, motor-language.

Adapted from Wyrwich KW, Schulz A, Nickel M, Slasor P, Ajayi T, Jacoby DR, et al. An adapted clinical measurement tool for the key symptoms of CLN2 disease. *Journal of Inborn Errors of Metabolism and Screening*. (2018) 6:2326409818788382. doi: 10.1177/2326409818788382. Published by Sage Journals, an open access article under the Creative Commons Attribution 4.0 License (https://creativecommons.org/licenses/by/4.0/).

**TABLE S2** **Baseline demographics and clinical characteristics of the overall cohort.**

|  | | **NH**  **(*N* = 52)** | **ERT-treated**  **(*N* = 24)** | ***p*-value** |
| --- | --- | --- | --- | --- |
| Sex, *n* (%) | Female | 25 (48) | 14 (58) | 0.41 |
|  | Male | 27 (52) | 10 (42) |  |
| Phenotype^a^, *n* (%) | Typical | 0 | 19 (79) | NA |
|  | Atypical | 0 | 4 (17) |  |
|  | Presymptomatic | 0 | 1 (4) |  |
|  | NA | 52 (100) | 0 |  |
| Genotype, *n* (%) | 2 common alleles^b^ | 29 (56) | 14 (58) | 0.70 |
|  | 1 common allele | 15 (29) | 5 (21) |  |
|  | 0 common alleles | 8 (15) | 5 (21) |  |
| Age at disease onset, years | Mean (SD) | *n* = 51  2.9 (0.8) | *n* = 23  3.4 (0.9) | 0.02 |
|  | Median (min, max) | 2.9 (1.0, 4.5) | 3.3 (2.0, 6.0) |  |
| Age at diagnosis, years | Mean (SD) | *n* = 45  4.7 (1.4) | 4.4 (2.1) | 0.54 |
|  | Median (min, max) | 4.5 (2.9, 9.8) | 4.2 (0.2, 9.5) |  |
| First symptom^c^, *n* (%) | Seizures | 31 (60) | 19 (79) | 0.09 |
|  | Language difficulties | 29 (56) | 16 (67) | 0.37 |
|  | Motor difficulties | 22 (42) | 4 (17) | 0.03 |
|  | Behavioral abnormalities | 11 (21) | 1 (4) | 0.06 |
|  | Dementia | 2 (4) | 1 (4) | 0.95 |
|  | Learning difficulties | 2 (4) | 1 (4) | 0.95 |
|  | Vision loss | 2 (4) | 0 | 0.33 |
|  | Other/unknown | 4 (8) | 1 (4) | NA |

^a^Phenotype was determined by physician adjudication.

^b^The common alleles were c.622C>T and c.509-1G>C.

^c^Patients may have had more than one presenting symptom.

ERT, enzyme replacement therapy; NA, not assessed; NH, natural history; SD, standard deviation.

**TABLE S3 Baseline demographics and clinical characteristics of NH and ERT-treated patients (sensitivity analysis: three-criteria match).**

|  | | **NH**  **(*N* = 18)** | **ERT-treated**  **(*N* = 18)** | ***p*-value** |
| --- | --- | --- | --- | --- |
| Sex, *n* (%) | Female | 7 (39) | 9 (50) | 0.50 |
|  | Male | 11 (61) | 9 (50) |  |
| Age at baseline, years | Mean (SD) | 4.4 (1.5) | 4.5 (1.5) | 0.81 |
|  | Median (min, max) | 4.4 (0.7, 8.5) | 4.4 (0.7, 8.5) |  |
| Baseline ML score | Mean (SD) | 4.0 (1.5) | 4.0 (1.5) | 1.00 |
|  | Median (min, max) | 4.0 (1.0, 6.0) | 4.0 (1.0, 6.0) |  |
| Baseline ML score category, *n* (%) | 1 | 1 (6) | 1 (6) |  |
|  | 2 | 2 (11) | 2 (11) |  |
|  | 3 | 3 (17) | 3 (17) |  |
|  | 4 | 6 (33) | 6 (33) |  |
|  | 5 | 2 (11) | 2 (11) |  |
|  | 6 | 4 (22) | 4 (22) |  |
| Phenotype^a^, *n* (%) | Typical | 0 | 15 (83) | NA |
|  | Atypical | 0 | 2 (11) |  |
|  | Presymptomatic | 0 | 1 (6) |  |
|  | NA | 18 (100) | 0 |  |
| Genotype, *n* (%) | 2 common alleles^b^ | 14 (78) | 14 (78) | 1.00 |
|  | 1 common allele | 3 (17) | 3 (17) |  |
|  | 0 common alleles | 1 (6) | 1 (6) |  |
| Age at disease onset, years | Mean (SD) | *n* = 17  2.9 (0.8) | *n* = 17  3.4 (0.8) | 0.06 |
|  | Median (min, max) | 3.0 (1.3, 4.4) | 3.3 (2.3, 6.0) |  |
| Age at diagnosis, years | Mean (SD) | *n* = 14  4.9 (1.6) | *n* = 18  4.0 (1.7) | 0.10 |
|  | Median (min, max) | 4.7 (2.9, 9.8) | 3.9 (0.2, 8.3) |  |
| First symptom^c^, *n* (%) | Seizures | 11 (61) | 16 (89) | 0.05 |
|  | Language difficulties | 6 (33) | 10 (56) | 0.18 |
|  | Motor difficulties | 8 (44) | 4 (22) | 0.16 |
|  | Behavioral abnormalities | 3 (17) | 0 | 0.07 |
|  | Learning difficulties | 0 | 1 (6) | 0.31 |
|  | Vision loss | 1 (6) | 0 | 0.31 |
|  | Other/unknown | 2 (11) | 1 (6) | NA |

^a^Phenotype was determined by physician adjudication.

^b^The common alleles were c.622C>T and c.509-1G>C.

^c^Patients may have had more than one presenting symptom.

Patients matched based on baseline age (± 12 months), baseline ML score (exact match), and genotype (0, 1, or 2 common alleles).

ERT, enzyme replacement therapy; ML, motor-language; NA, not assessed; NH, natural history; SD, standard deviation.

**TABLE S4** **Rate of decline in ML score** **for NH and ERT-treated patients (sensitivity analysis: three-criteria match).**

|  | **NH**  **(*N* = 18)** | **ERT-treated  (*N* = 18)** |
| --- | --- | --- |
| Rate of decline in ML score, points per 48 weeks | | |
| Mean (SD) | 2.07 (1.61) | 0.49 (0.43) |
| Median (min, max) | 2.18 (0.00, 5.60) | 0.47 (0.00, 1.33) |
| 95% CI | 1.27, 2.87 | 0.28, 0.70 |
| Mean (SE) difference | 1.58 (0.39) | |
| 95% CI | 0.76, 2.40 | |
| *p*-value | 0.0007 | |

Patients matched based on baseline age (± 12 months), baseline ML score (exact match), and genotype (0, 1, or 2 common alleles).

CI, confidence interval; ERT, enzyme replacement therapy; ML, motor-language; NH, natural history; SD, standard deviation; SE, standard error.


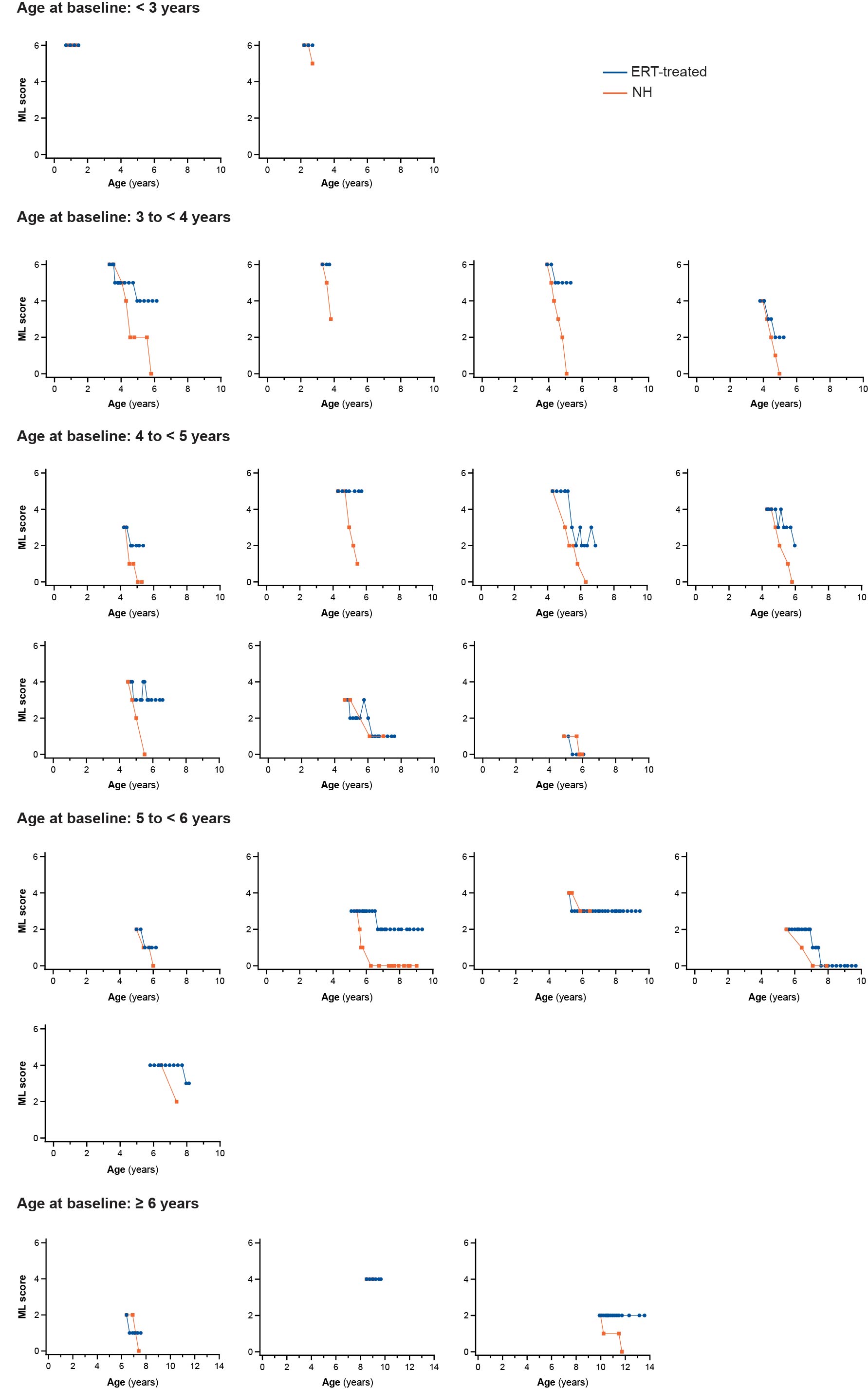


**FIGURE S1**

ML score over follow-up time for matched-patient pairs grouped by baseline age (two-criteria match). Patients matched based on baseline age (± 12 months) and baseline ML score (exact match). ERT, enzyme replacement therapy; ML, motor-language; NH, natural history.


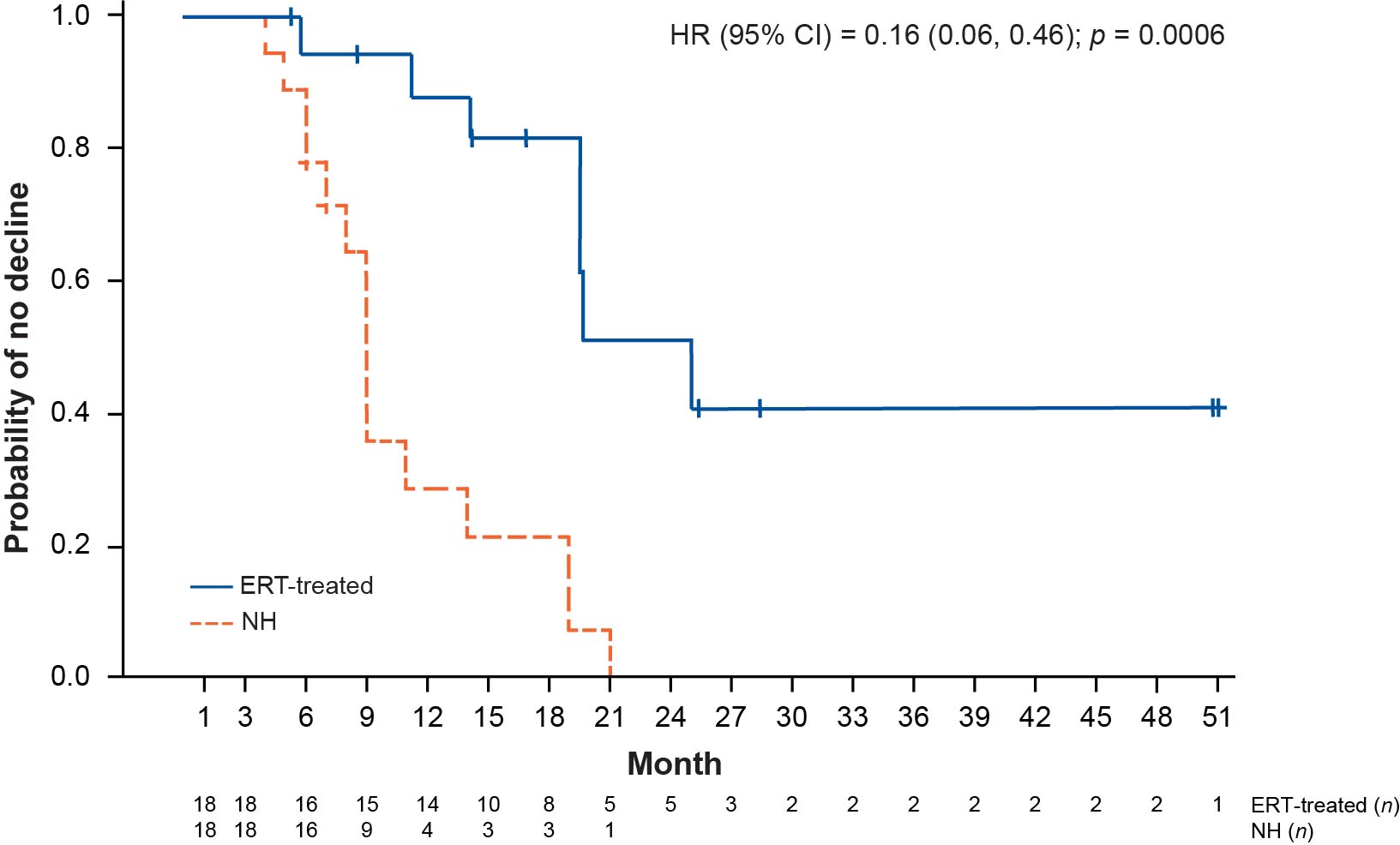


**FIGURE S2**

Kaplan–Meier plot of time to unreversed 2-point decline or score of 0 in ML domains for NH and ERT-treated patients (sensitivity analysis: three-criteria matched cohorts). Patients matched based on baseline age (± 12 months), baseline ML score (exact match), and genotype (0, 1, or 2 common alleles). CI, confidence interval; ERT, enzyme replacement therapy; HR, hazard ratio; ML, motor-language; NH, natural history.


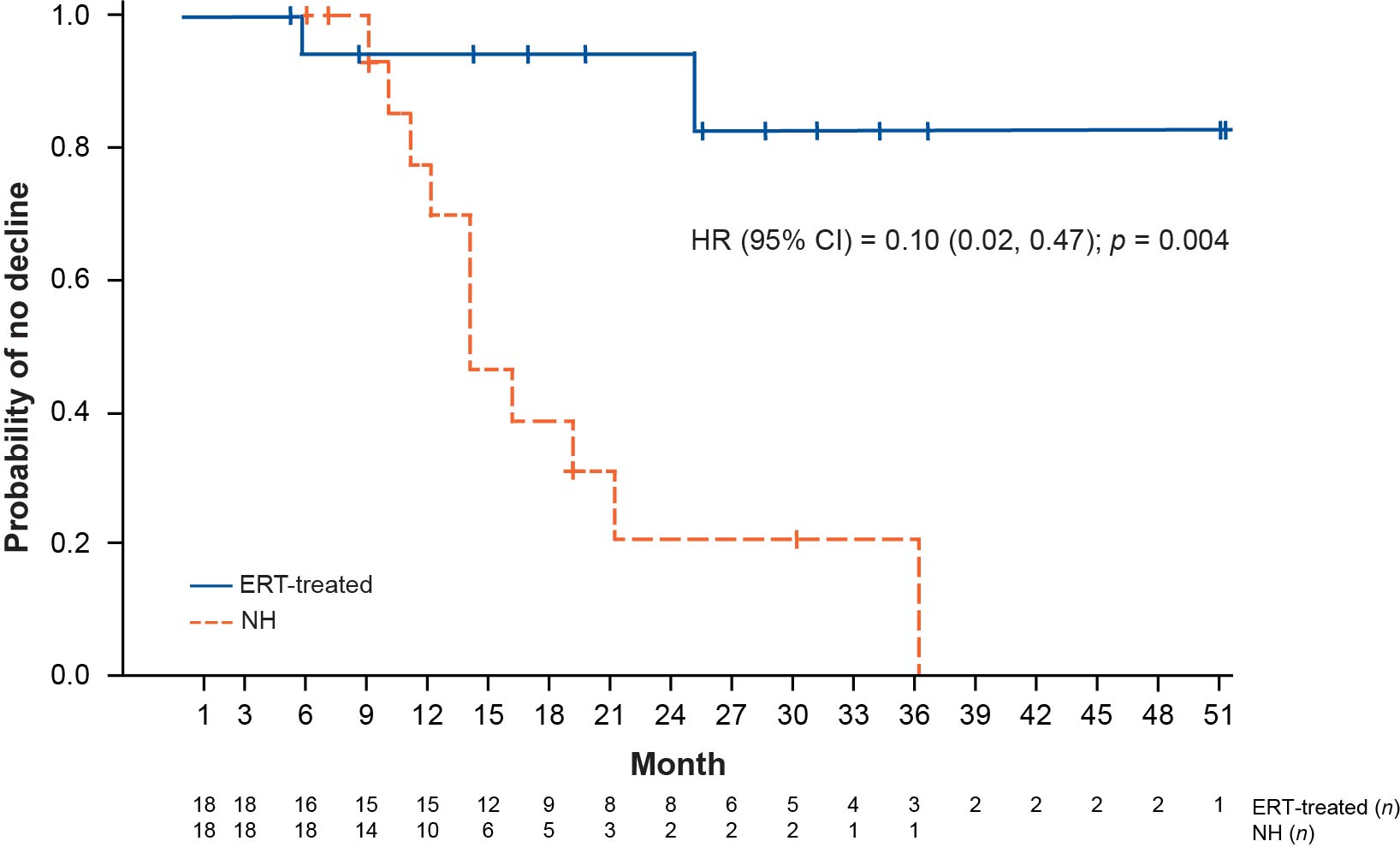


**FIGURE S3**

Kaplan–Meier plot of time to unreversed score of 0 in ML domains for NH and ERT-treated patients (sensitivity analysis: three-criteria matched cohorts). Patients matched based on baseline age (± 12 months), baseline ML score (exact match), and genotype (0, 1, or 2 common alleles). CI, confidence interval; ERT, enzyme replacement therapy; HR, hazard ratio; ML, motor-language; NH, natural history.
